# Effect of COVID-19 vaccine on blood glucose metrics in Arabic people with type 1 diabetes

**DOI:** 10.3389/fendo.2023.1120384

**Published:** 2023-03-20

**Authors:** Ebaa Al-Ozairi, Mohammad Irshad, Etab Taghadom, Anisha Varghese, Litty Sojan, Jumana Alkandari

**Affiliations:** ^1^ DAFNE Unit, Dasman Diabetes Institute, Kuwait City, Kuwait; ^2^ Department of Medicine, Faculty of Medicine, Kuwait University, Safat, Kuwait; ^3^ Al-Amiri Hospital, Ministry of Health, Kuwait City, Kuwait

**Keywords:** COVID-19 vaccine, type 1 diabetes, blood glucose metrics, time in range (TIR), glucose variability, SARS- CoV-2

## Abstract

**Introduction:**

People with diabetes are at a higher risk for coronavirus disease-19 (COVID-19) and hence are prioritized for vaccination. The aim of the current study was to investigate the effects of COVID-19 vaccination on blood glucose control in Arabic people with type 1 diabetes (T1D). Secondary aim was to compare the responses between the two vaccines approved for use in Kuwait.

**Method:**

This retrospective study compared ambulatory glucose metrics, using a continuous glucose monitoring device, measured for 14 days before, and 7 days and 14 days after, the first and second dose of the COVID-19 vaccine in Arabic people with Type 1 diabetes (T1D). We also explored possible links with vaccine type and other clinical characteristics. Glucose metrics calculated were time in range (TIR, 3.9–10 mmol/L), time above range (TAR, 10.1- 13.9 mmol/L or >13.9 mmol/L), time below range (TBR, 3- 3.9 mmol/L or <3 mmol/L) and glucose variability (CV).

**Results:**

We enrolled 223 participants in the study. Over the 7 days period after the first vaccination dose there was a decrease in TIR (mean difference (SD) –1.9% ± 14.8%; p = 0.05) and increase in TAR >10 mmol/L (2.2% ± 15.9%; p = 0.04), with no effects on TBR. These effects were not seen after the second dose or 14 days after either dose. There was a decrease in CV over the 7 days period after the first (−1.2% ± 7.4%; p = 0.02) and second vaccine doses (−1.1% ± 6.9%; p = 0.03), with no effects noted 14 days after either dose. In subgroup analysis similar effects on TIR and TAR were also seen in those who had received the viral vector-based vaccine, but not the mRNA-based vaccine, although the decrease in CV was seen in those who had received the mRNA based vaccine but not the viral vector-based vaccine.

**Conclusion:**

We found a temporary impairment in glucose control in the first 7 days, particularly among individuals receiving viral vector vaccines. The group receiving mRNA vaccine was likely to experience an increase in glucose levels above the target range. However, the temporary change in metrics appears to return to pre-vaccination levels after one-week post-vaccination. The effects on glycemic parameters were more neutral after the second dose.

## Introduction

People with diabetes mellitus are more susceptible to severe acute respiratory syndrome coronavirus-2 (SARS- CoV-2) infection than those without diabetes (1–3). The morbidity and mortality of coronavirus disease (COVID-19) were significantly high in people with diabetes compared to those without diabetes (4, 5). Infected with SARS-CoV-2 can present with severe illness and have a poor prognosis (2, 3). The only way to slow the rapid spread of the infection was to immunize the global population. The pharmaceutical industry collaborated with research groups to develop effective vaccines in a short span of time (6–8). Diabetes patients were given the COVID-19 vaccine on priority (2, 3). However, the reluctance to vaccinate among individuals with diabetes was high owing to a fear of diabetes-related adverse events (9). Vaccination against SARS-CoV-2 has undoubtedly played a critical role in preventing infection and lowering morbidity and mortality rates (10).

T1D is a chronic autoimmune disease that requires patients to undergo lifelong exogenous insulin therapy because of the immune-mediated destruction of insulin-secreting pancreatic beta cells (4). It was presumed that the vaccine against SARS-CoV-2 stimulates an immunological response among people with diabetes there has been some data to indicate that the COVID-19 vaccine may alter blood glucose regulation, in people with T1D, due to its downstream effects on metabolism (11). Furthermore, several proinflammatory cytokines are produced during the cellular and humoral immune response to the vaccine (12), and these cytokines may increase insulin resistance (11, 13). Therefore, management of sub-optimal glycemic levels that occur soon after vaccination is necessary since they may substantially affect the longevity or potency of immunity in people with diabetes (14).

Many case studies have reported the side effects of the novel coronavirus vaccine in people with T1D, including nausea, vomiting, and local or systemic inflammation (15, 16). Some case studies observed symptoms of hyperglycemia, diabetic ketoacidosis (DKA), and hyperosmolar hyperglycemic state (17, 18). There has yet, however, been a detailed investigation into the effects of the vaccine on blood glucose control in people with T1D. The majority of people with T1D residing in Kuwait have received two doses of COVID-19 vaccines. The Ministry of Health, Kuwait, has authorized the mRNA-based vaccine produced by Pfizer-BioNTech^®^ and the viral vector-based vaccine produced by Oxford-AstraZeneca^®^ for vaccination. In clinical practice, we noticed perturbations in blood glucose metrics in people with T1D who had received the COVID-19 vaccines but this was anecdotal and not systematically investigated. The aim of the current study, therefore, was to investigate the effects of COVID-19 vaccination on blood glucose control in Arabic people with T1D. A secondary aim was to compare the responses between the two vaccines approved for use in Kuwait.

## Materials and methods

### Data collection and procedures

This is a retrospective observational study. We collected continuous glucose monitoring data of people with T1D who regularly visited the Dose Adjustment for Normal Eating (DAFNE) clinic at Dasman Diabetes Institute, Kuwait. All participants used the Libre View^®^ flash glucose monitor (FGM) device for blood glucose monitoring. Those with partial (<70% of study period) or no FGM data were excluded from this study. All participants were interviewed and information on COVID-19 infection, vaccination date, type of vaccine given, and adverse events was collected. Adverse events were considered serious when they threatened the survival or physical integrity of the patients or required hospitalization. We also collected data on the diabetes duration, age, HbA1c, and vitals from electronic health records. Body mass index (BMI) was calculated as weight divided by height squared (kg/m2). All participants had received two doses of the COVID-19 vaccine. The study was performed per the Helsinki Declaration’s principles and approved by the Institutional Review Board (435/2016), with informed consent.

FGM blood glucose data were collected 14 days before a vaccine dose and 7, and 14, days after both vaccine doses. Glucose metrics calculated included percentage of glucose variability (CV), blood glucose metrics falling within target ranges (time in range (TIR), 3.9–10 mmol/L), above the target range (time above the range (TAR >13.9 mmol/L and 10.1 – 13.9 mmol/L), and below the target range (time bellow the range (TBR 3.0 -3.8 mmol/L and TBR < 3.0 mmol/L).

### Statistical analysis

SPSS Statistics^®^ version 29 was used for all statistical analyses. Data were expressed as the mean ± standard deviation (SD), median with range, and frequency where appropriate. The Kolmogorov–Smirnov test was used to assess the normality of data. Student’s paired t-tests were used to compare means for data with a normal distribution; otherwise, the Wilcoxon Signed Ranks test was used. The effect size was calculated through Cohen’s d, and values 0.20, 0.40, and 0.60 indicate small, medium, and large effect sizes, respectively (19, 20).

### Results

**Table 1** presents the basic information of the participants. The mean age was 32.6 ± 9.1 years, the diabetes duration was 16.8 ± 8.1 years, 53.4% were female and 24.7% of them were infected with COVID-19. The HbA1c range was 4.9%–13.8% (median: 7.8%), and 60.5% had HbA1c > 7%. The mean BMI was 27.3 ± 4.9 kg/m (2), and 24.7% of participants were obese. All participants were on multi-daily dose injections, using long-acting and rapid-acting insulin medication.

**Table 1:** Baseline information of the people with type 1 diabetes.

	Total (n= 223)	Viral vector-based vaccine (n=90)	mRNA- based vaccine (n=133)	
	Mean ± SD or n (%)	Mean ± SD or n (%)	Mean ± SD or n (%)	p-value*
Age (years)	32.6 ± 9.1	34.7 ± 9.5	31.2 ± 8.6	0.005
Gender				
Male	104 (46.6)	45 (50.0)	59 (44.4)	0.41
Female	119 (53.4)	45 (50.0)	74 (55.6)	
Diabetes duration (years)	16.8 ± 8.1	17.5 ± 8.1	16.4 ± 8.2	0.32
HbA1c (%)	7.9 ± 1.3	7.9 ± 1.3	8.0 ± 1.2	0.58
BMI (kg/m2)	27.3 ± 4.9	27.6 ± 5.1	27.1 ± 5.1	0.52
Systolic BP (mmHg)	119.0 ± 11.9	118.7 ± 12.5	119.1± 11.6	0.82
Diastolic BP (mmHg)	73.7 ± 8.7	73.7 ± 9.1	73.7 ± 8.5	0.99
TAR (%)	39.8 ± 19.6	37.9 ± 20.6	41.0 ± 18.8	0.25
TIR (%)	55.9 ± 18.2	57.8 ± 19.0	54.5 ± 17.6	0.18
TBR (%)	4.5 ± 4.6	4.5 ± 5.1	4.5 ± 4.3	0.99
CV (%)	37.1 ± 7.4	36.2 ± 7.5	37.6 ± 7.3	0.17
Normal BMI (≤ 24.9 kg/m2)	69 (30.9)	21 (23.3)	48 (36.1)	0.04
Overweight (25- 29.9 kg/m2)	99 (44.4)	49 (54.4)	49 (54.4)	
Obese (> 29.9 kg/m2)	55 (24.7)	20 (22.2)	35 (26.3)	
HbA1c ≤ 7.5%	88 (39.5)	38 (42.2)	50 (37.6)	0.49
HbA1 >7.5%	135 (60.5)	52 (57.8)	83 (62.4)	
COVID-19 infection, Yes	55 (24.7)	18 (20)	37 (27.8)	0.18
No	168 (75.3)	72 (80.0)	96 (72.2)	
Basal insulin dose (U/day)	23.7 ± 10.3	23.8 ± 9.0	23.7 ± 11.1	0.90
Bolus insulin dose (U/day)	29.5 ± 14.9	31.1 ± 16.0	28.6 ± 14.3	0.35
Daily insulin dose (U/day)	52.1 ± 21.6	53.8 ± 21.5	51.1 ± 21.6	0.48

*Independent t-test used for continuous variables and Chi-square test used for categorical variables. Glucose metrics represents, % time above the range (TAR) >10.1 mol/L; % time in range (TIR), 3.9–10 mmol/L; % time bellow the range (TBR), <3.9mmol/L and % glucose variability (CV).

Before vaccination, the mean interstitial glucose on target range (TIR) was 55.9% ± 18.2%. However, 7 days after the first vaccine dose, TIR significantly decreased (mean differences (SD) –1.9% ± 14.8%; *p* = 0.05). This phenomenon was mirrored by an increase in interstitial glucose levels above the target range (TAR > 10 mmol/L). Before vaccination, the mean TAR was 39.8% ± 19.6%; after the first dose, the TAR significantly increased (2.2% ± 15.9%, p = 0.04). Over the 7-day period following the second dose, there was no effect on TIR (−1.6% ± 16.4%, *p* = 0.17) or TAR (1.5% ± 16.2%, p= 0.21) (**Table 2**). The proportion of people who experienced an increase of >10% in TAR (> 10 mmol/L) after the first and second doses was equal (26.9%). The result also showed a significant decrease in CV during 7 days after the first (−1.2% ± 7.4%, p = 0.02) and second vaccine doses (−1.1% ± 6.9%, p = 0.03).

**Table 2:** Changes in glucose metrics (%) after COVID-19 vaccine in people with type 1 diabetes.

.	First dose				Second dose			
Glucose metrics	7 days	d†	14 days	d†	7 days	d†	14 days	d†
	MD ± SD		MD ± SD		MD ± SD		MD ± SD	
TAR (>10 mmol/L)	12.2 ± 15.9*	0.14	-0.2 ± 12.5	-0.01	1.5 ± 16.2	0.09	0.003 ± 13.7	0.00
TIR (3.9–10 mmol/L)	-1.9 ± 14.8*	-0.13	0.04 ± 11.6	0.00	-1.6 ± 16.4	-0.10	-0.7 ± 11.9	-0.06
TBR (< 3.8 mmol/L)	-0.4 ± 4.4*	-0.10	0.04 ± 4.3	0.01	-0.2 ± 5.1	-0.05	0.05 ± 3.9	0.01
CV	-1.2± 7.4*	-0.17	-0.3± 6.4	-0.04	- 1.1± 6.9*	-0.16	0.2± 6.1	0.03

Wilcoxon Signed Ranks Test* p <0.05, †Effect size pre-posttest. MD- mean difference, SD- standard deviation. Glucose metrics presents, % time above the range (TAR); % time in range (TIR); % time below range (TBR) and % glucose variability (CV).

We also evaluated glucose metrics after 14 days after vaccination. The result show that small insignificant TIR changes occurred after the first (0.04% ± 11.6%) and second (−0.7% ± 11.9%) vaccine doses. Similarly, the changes in TAR and CV changes were also small and insignificant14 days after the first and second vaccine doses. The effect size from pre- to post-test after 7 days and 14 days of vaccination indicated a small effect of vaccine dose on the glucose metrics (d ≤ 0.17).

During the entire vaccination cycle, no significant changes were noted in terms of insulin dose (p = 0.78), HbA1c levels (p = 0.42), BMI (p = 0.57), and systolic and diastolic blood pressure (p ≥ 0.61).

### Subgroup analysis

We compared the glucose metrics in people with T1D who received the viral vector-based vaccine (n = 90) and mRNA vaccine (n = 133). Both groups had almost similar sex distribution, cases of COVID-19 infection, mean diabetes duration, BMI, HbA1c, insulin dose, and glucose metrics (**Table 1**).

### Viral vector-based vaccine

After 7 days of the first dose of viral vector-based vaccine, TIR significantly decreased (−4.2% ± 13.4%, p = 0.001), whereas the TAR ranging from 10-13.9 mmol/L increased (3.7% ± 10.6%, p = 0.001). The lower HbA1c (≤ 7%) group showed a significant decrease in TIR (−5.4% ± 11.8%, p = 0.001) compared to the higher HbA1c (>7%) group (−3.4% ± 14.6%, p = 0.10) (**Table 3**). Remarkably, the changes in glycemic metrics among the individuals affected by SARS-CoV-2 were insignificant. People with Normal-BMI exhibited a significant increase in the TAR range of 10 - 13.9 mmol/L (8.3% ± 12.2%, p = 0.001) and decreased in TIR (−8.2% ± 15.8%, p = 0.001). However, obese people showed an increase in TAR >13.9 mmol/L level (2.5% ± 9.3%, p= 0.25) compared to the overweight and normal- BMI people.

**Table 3:** Changes in glucose metrics (%) after 7 days of viral vector-based and mRNA-based COVID-19 vaccine in people with type 1 diabetes.

	First Dose						Second Dose					
Viral vector-based vaccine	TAR-L1	TAR-L2	TIR	TBR-L1	TBR-L2	CV	TAR-L1	TAR-L2	TIR	TBR-L1	TBR-L2	CV
	MD± SD	MD± SD	MD± SD	MD± SD	MD± SD	MD± SD	MD± SD	MD± SD	MD± SD	MD± SD	MD± SD	MD± SD
**All**	0.5± 10.2	3.7± 10.6**	-4.2± 13.4**	-0.2± 3.1	0.1± 2.0	-1.2± 7.5	-0.6± 9.4*	1.9± 9.8	-1.6± 13.3	0.0± 3.6	0.2± 2.7	-0.1± 6.7
COVID-19 infection, Yes	1.1± 7.8	-2.8± 9.6	1.0± 8.8	0.5± 3.7	0.2± 1.3	1.4± 7.5	-0.6± 12.4	1.1± 11.4	-1.1± 15.3	-0.1± 1.8	-0.1± 0.6	-0.1± 6.1
** **No	0.4± 10.7	5.2± 10.3**	-5.5± 14.1**	-0.4± 3.0	0.0± 2.1	-1.8± 7.5*	-0.6± 8.6	2.1± 9.4*	-1.8± 12.8	0.0± 3.9	0.3± 3.0	-0.1± 6.9
HbA1c ≤ 7%	1.8± 5.0	4.1± 9.0**	-5.4± 11.8**	-0.5± 3.6	0.0± 2.2	-0.7± 8.2	0.2± 5.1	1.6± 8.2	-1.4± 12.6	-0.6± 4.4	-0.1± 1.9	0.5± 7.5
HbA1c > 7%	-0.5± 12.7	3.4± 11.7	-3.4± 14.6	-0.1± 2.8	0.1± 1.8	-1.6± 7.1	-1.2± 11.6	2.2± 10.9	-1.8± 13.9	0.4± 2.9	0.4± 3.2	-0.6± 6.1
Normal BMI (≤ 24.9 kg/m2)	1.7± 7.7	8.3± 12.2**	-8.2± 15.8**	-1.4± 3.4	-0.4± 1.5	-2.6± 5.5	-1.7± 8.1	0.8± 7.9	1.4± 11.0	-0.5± 3.8	-0.1± 1.6	-0.3± 4.0
Overweight (25- 29.9 kg/m2)	-0.8± 11.4	3.1± 10.0*	-3.0± 13.0	0.3± 3.2	0.4± 1.9	-0.3± 8.1	-0.5± 10.6	2.5± 11.1*	-2.7± 15.0	0.4± 3.5	0.0± 1.4	0.4± 7.9
Obese (29.9 kg/m2)	2.5± 9.3	0.6± 9.4	-3.3± 11.8	-0.6± 2.3	-0.2± 2.4	-2.2± 7.8	0.1± 7.0	1.4± 7.8	-1.9± 9.8	-0.6± 3.6	1.0± 5.4	-1.3± 5.6
mRNA- based vaccine												
**All**	2.0± 12.0	-0.8± 10.0	-0.4± 15.5	-0.4± 3.4	-0.2± 1.7*	-1.2± 7.4*	1.9± 13.2	-0.4± 11.3	-1.6± 18.4	-0.3± 4.1	-0.3± 1.9	-1.8± 7.0**
COVID-19 infection, Yes	1.3± 10.8	-0.1± 8.5	-0.4± 15.5	-0.6± 2.7	-0.2± 1.6	-0.5± 7.5	3.8± 19.5	-1.6± 12.7	-2.0± 19.3	0.3± 5.2	-0.5± 2.0	-3.0± 7.6*
No	2.2± 12.5	-1.1± 10.5	-0.4± 15.6	-0.4± 3.7	-0.1± 1.8	-1.5± 7.4*	1.3± 10.3	0.1± 10.8	-1.5± 18.2	-0.5± 3.7	-0.2± 1.8	-1.4± 7.1*
HbA1c ≤ 7%	0.7± 6.9	0.4± 8.7	-1.0± 11.7	-0.2± 3.8	0.2± 2.4	0.2± 7.2	2.9± 10.4	0.0± 10.2	-3.5± 19.3	-0.4± 5.1	-0.4± 2.1	-2.5± 7.5*
HbA1c > 7%	2.8± 14.2*	-1.5± 10.7	0.0± 17.4	-0.6± 3.2	-0.3± 1.0**	-2.1± 7.5*	1.3± 15.0	-0.6± 12.1	-0.3± 17.8	-0.2± 3.3	-0.2± 1.6	-1.3± 7.6*
Normal BMI (≤ 24.9 kg/m2)	1.0± 10.0	0.8± 10.0	-1.7± 15.2	-0.1± 3.6	0.0± 2.2	-1.1± 7.0	0.1± 8.8	0.8± 10.3	-0.3± 15.5	-0.3± 4.0	-0.4± 2.1	-1.9± 7.7
Overweight (25- 29.9 kg/m2)	2.7± 12.8	-2.2± 10.3	0.6± 15.8	-0.3± 3.6	-0.4± 1.3	-0.7± 7.8	2.3± 12.2	-0.9± 12.6	-2.3± 22.3	-0.4± 4.0	-0.4± 2.0	-1.9± 7.3
Obese (> 29.9 kg/m2)	2.3± 13.4	-1.1± 9.5	0.1± 15.8	-1.0± 2.9*	-0.1± 1.4	-2.3± 7.1	4.4± 19.9	-1.6± 10.8	-2.8± 15.7	0.0± 4.7	0.0± 1.1	-1.4± 7.1*

Wilcoxon Signed Ranks Test* p <0.05, **p <0.01, MD, mean difference; SD, standard deviation. Glucose metrics presents, % time above the range in level 1 (TAR-LI) >13.9 mmol/L and level 2 (TAR-L2), 10.1 – 13.9 mmol/L; % time in range (TIR), 3.9–10 mmol/L; % time bellow the range in level 1 (TBR-L1), 3.0 -3.8 mmol/L, and level 2 (TBR-L2) < 3.0 mmol/L and % glucose variability (CV).

After 7 days of the second dose, the decrease in TIR and increase in TAR were insignificant (**Table 3**). Also, no significant changes were found in the lower HbA1c and non-obese groups. After the first and second doses, many people increased TAR by more than 10% in the range of 10–13.9 mmol/L (24.4% and 21.1%) and >13.9 mmol/L (12.2% and 8.9%), respectively. However, after 14 days of first and second doses, the changes in glucose metrics were minor and insignificant (**Supplementary Table 1**).

### mRNA-base vaccine

After 7 days of the first and second doses of mRNA vaccine administration insignificant changes were noted in the glucose metrics (**Table 3**). There was a significant decrease in CV after the first (−1.2% ± 7.4%, p = 0.06) and second (−1.8% ± 7.0%, p = 0.01) doses. The decrease in TAR and TBR among SARS-CoV2-infected and non-infected people was almost equal and insignificant. However, those with higher HbA1c levels exhibited a significant decrease in TBR < 3.0 mmol/L levels (- 0.3% ± 1.0%, p= 0.01) and increase in TAR >13.9 mmol/L (2.8% ± 11.2%, p= 0.05). Obese and overweight people also exhibited a an increase in TAR >13.9 mmol/L levels after the first and second doses (**Table 2**). After 7 days of the first and second doses, a greater than 10% in TAR 10–13.9 mmol/L (12.0% and 13.5%) and >13.9 mmol/L (15.0% and 16.5%) were noted respectively. Further 14 days after receiving the first and second doses, the differences in glucose metrics were reduced (**Supplementary Table 1**).

### Adverse events

No patients experienced severe hyperglycemia, hypoglycemia, and diabetes ketoacidosis throughout the vaccination periods.

## Discussion

This study evaluated the glucose metrics among Arab people with TID immediately after receiving COVID-19 vaccination. We examined the change in glucose metrics at 7 and 14 days after receiving the first and second doses of vaccines. We found a small but significant decrease in the time in target range, an increase in glucose levels above the target range, and a decrease in glycemic variability during the 7 days period after the first dose. No effects of vaccination were found after the second dose. These findings appear to be primarily driven by the viral vector-based, and not the mRNA based, vaccine.

These results indicated a temporary worsening of glycemic control that lasted for 1 week after vaccination. The differences in glucose metrics after the first dose was consistent with those reported in several studies (21–24). In accordance with our results, these previous studies reported a decrease in TIR and CV among those with TID at 3 or 7 days after receiving the first dose of vaccine (21–24). However, these studies have minor disparities in terms of the percent changes in glucose metrics and variability. This could be because of the vaccine type, study setting, and devices used for glucose monitoring. In our study, TIR reduction was greater in the group that received viral vector-based vaccines than those who received mRNA-based vaccines. These results were consistent with several studies wherein the decrease in TIR was mild in people with T1D who received the mRNA-based vaccine (15, 21, 24). The mild effect of the mRNA vaccine on the glucose profile was also reported in children and adolescents with TID (24, 25). The precise reasons for these change in glucose metrics after vaccination are yet to be identified. Further, the fluctuation in blood glucose levels may be multifactorial. According to some theories, vaccine-induced immune response may cause inflammation, thus disrupting insulin sensitivity and glucose metabolism (26). Malaise after vaccination may also disrupt insulin treatment, resulting in impaired glucose control (18). Furthermore, epinephrine and cortisol hormone levels increase in response to stress after vaccination, which may further increase blood glucose levels (10, 27).

A previous study reported a more pronounced perturbations in glucose metrics in people with lower glycemic (HbA1c) levels, which is consistent with our findings (11). No other study has correlated the glucose metrics with vaccine type or glycemic levels. To the best of our knowledge, this is the first study to report the perturbation of glucose metrics in SARS-CoV-2-infected people with T1D. The result showed worse but temporary perturbation of glucose metrics in infected people who received the viral vector-based vaccine. Our result shows more increase in glucose levels above the target range after vaccination with the mRNA vaccine. Even after the second dose, this effect was stronger in people with higher glycemic levels, overweight and obese people with T1D. In a study of people with T1D, glucose metrics were measured immediately after the first and second doses of the mRNA COVID-19 vaccine (28). Similar to our study, they also found an insignificant decrease in TIR and an increase of TAR after the first and second dose of vaccination (28).

Most studies focused on the perturbation of glucose on the target range and glycemic variability. However, the increase in glucose above the target range cannot be ignored. Acute hyperglycemia in people with diabetes can lead to life-threatening complications such as diabetic ketoacidosis and hyperosmolar hyperosmotic state (29). Regardless of the vaccine type, possible vaccination-induced hyperglycemia and associated complications are reported among people with diabetes (30). In the present study, blood sugar increased above the target range in both vaccinated groups. The mRNA vaccination group on the other hand experienced an increase in glucose variability. The mRNA vaccines may encode the SARS-CoV-2 full-length spike which causes the same mechanism of hyperglycemia as the virus infection (29, 31, 32). Short-term increase in blood glucose levels due to the inflammatory response after administering a vaccine against the SARS-CoV-2 virus is common (30). However, immediate management of hyperglycemia following vaccination is critical because prolonged hyperglycemia can affect the durability and strength of immunity (14). There is evidence that sub-optimal glycemic control boost the immune response significantly (33).

During the 14 days after vaccination, glucose metrics recovered to the previous metrics. A study found a higher frequency of hyperglycemia, poor diabetes control, and hypoglycemia after the COVID-19 vaccination (32). Some cases also report DKA episodes after vaccination (17, 18). However, in the present study, we did not find DKA, severe hypoglycemia, or hypoglycemic events during the vaccination cycle. The lack of severe post-vaccination events were reported in this study; this could be because all study participants were DAFNE graduates who had received structured education and had learned to manage adverse diabetes events on time (34). In addition, all participants were using continuous glucose monitoring device that provides real-time information on blood glucose levels, which can be used to adjust insulin dosing and diet. The use of such a device may help prevent severe episode of hypoglycemia (35). Our findings suggest that people with T1D should undergo counselling and be prepared for possible hyperglycemia immediately after receiving mRNA -based vaccination. Our findings could help clinicians and caregivers to be aware of possible severe hyperglycemia and its complications.

The main limitation of this study was that all participants were DAFNE graduates delivered in one site. The small sample size also limits the application of sub-group findings to large population.

## Conclusions

Temporary but small perturbations in glucose metrics were observed immediately after receiving the first dose of COVID-19 vaccination. This effect was more prominent in the group that received the viral vector-based vaccine. However, the group receiving the mRNA vaccine is likely to exhibit increased glycose metrics above the target range, particularly those with poor glycemic control and obesity. However, this alteration of metrics is temporary and comes back to previous levels after one-week post-vaccination. Further, the effects on glycemic parameters were even more neutral after the second dose.

## Data availability statement

The original contributions presented in the study are included in the article/**Supplementary Material**. Further inquiries can be directed to the corresponding author.

## Ethics statement

The studies involving human participants were reviewed and approved by Ethical Committee of Dasman Diabetes Institute, Kuwait. The patients/participants provided their written informed consent to participate in this study.

## Author contributions

Conceptualization, EO, ET and JA. Data acquisition, AV and LS. Analysis and writing- original draft, MI. Critical review and editing, EO. All authors contributed to the article and approved the submitted version.
