# Supplementary material for: Effect of COVID-19 vaccine on blood glucose metrics in Arabic people with type 1 diabetes
Source: Front Endocrinol (Lausanne). 2023 Mar 20;14:1120384. doi: 10.3389/fendo.2023.1120384 (PMC10067894; doi:10.3389/fendo.2023.1120384)
Supplement: Supplementary file 1 [file Table_1.docx]

Supplementary table 1: Changes in glucose metrics (%) after 14 days of viral vector-based and mRNA-based COVID-19 vaccines in people with type 1 diabetes.

|  | First dose | |  |  |  | Second dose | |  |  |  |
| --- | --- | --- | --- | --- | --- | --- | --- | --- | --- | --- |
| Viral vector-based vaccine | TAR (L1) | TAR (L2) | TIR | TBR (L1) | TBR (L2) | TAR (L1) | TAR (L2) | TIR | TBR (L1) | TBR (L2) |
|  | MD± SD | MD± SD | MD± SD | MD± SD | MD± SD | MD± SD | MD± SD | MD± SD | MD± SD | MD± SD |
| All | -0.1± 11.2 | 1.2± 7.9 | -1.4± 11.7 | 0.1± 2.9 | 0.1± 2.5 | -1.0± 7.5 | 0.4± 9.1 | -0.3± 11.8 | 0.3± 3.2 | 0.3± 2.6 |
| COVID-19 infection, Yes | -1.4± 10.1 | -1.9± 7.6 | 2.3± 10.2 | 0.7± 3.7 | 0.3± 1.0 | -1.8± 9.4 | 0.4± 8.9 | 0.4± 13.5 | 0.7± 2.1 | 0.3± 1.2 |
| No | 0.2± 11.5 | 2.0± 7.9* | -2.4± 12.0* | -0.1± 2.7 | 0.0± 2.7 | -0.8± 7.1 | 0.4± 9.2 | -0.4± 11.4 | 0.3± 3.4 | 0.3± 2.9 |
| HbA1c ≤ 7% | 1.3± 4.7 | 1.1± 7.1 | -2.0± 8.9 | 0.0± 3.1 | -0.3± 1.7 | 0.3± 4.6 | 0.2± 8.2 | -0.6± 11.7 | 0.1± 3.7 | -0.1± 1.4 |
| HbA1c > 7% | -1.1± 14.1 | 1.3± 8.6 | -1.0± 13.5 | 0.2± 2.8 | 0.3± 2.9 | -2.1± 9.1 | 0.5± 9.8 | 0.0± 12.0 | 0.6± 2.7 | 0.6± 3.3 |
| Normal BMI (≤ 24.9 kg/m2) | -0.3± 8.5 | 2.2± 8.8 | -1.3± 15.4 | -0.4± 3.2 | -0.3± 1.6 | -1.4± 7.8 | -0.9± 8.4 | 2.9± 11.4 | -0.1± 2.7 | -0.5± 1.2 |
| Overweight (25- 29.9 kg/m2) | -0.8± 12.7 | 1.7± 8.4 | -1.3± 11.7 | 0.4± 3.1 | 0.0± 1.3 | -0.9± 7.6 | 0.8± 9.7 | -0.8± 12.5 | 0.7± 3.6 | 0.2± 1.6 |
| Obese (29.9 kg/m2) | 1.6± 9.6 | -1.1± 5.4 | -1.8± 7.4 | -0.2± 2.2 | 0.5± 4.7 | -0.8± 7.6 | 0.5± 8.3 | -1.9± 10.1 | -0.1± 2.2 | 1.3± 4.7 |
| mRNA- based vaccine | |  |  |  |  |  |  |  |  |  |
| All | -0.7± 8.6 | -0.3± 8.3 | 1.0± 11.4 | -0.1± 3.1 | 0.1± 2.1 | 0.1± 9.7 | 1.3± 8.4 | -1.0± 12.1 | -0.2± 2.7 | -0.1± 1.3 |
| COVID-19 infection, Yes | -0.4± 9.5 | 0.6± 7.0 | 0.2± 11.6 | -0.2± 2.6 | -0.2± 1.1 | 0.0± 10.9 | 1.2± 9.2 | -1.3± 12.4 | 0.4± 2.8 | -0.2± 1.4 |
| No | -0.8± 8.3 | -0.7± 8.7 | 1.4± 11.4 | -0.1± 3.3 | 0.2± 2.3 | 0.1± 9.2 | 1.3± 8.1 | -0.9± 12.0 | -0.4± 2.6 | -0.1± 1.2 |
| HbA1c ≤ 7% | -0.4± 4.9 | 0.0± 7.0 | 0.0± 8.4 | 0.0± 3.4 | 0.5± 2.6 | 1.2± 7.8 | 1.7± 7.0 | -2.5± 11.5 | -0.2± 2.6 | -0.1± 1.3 |
| HbA1c > 7% | -0.8± 10.2 | -0.5± 9.0 | 1.7± 12.9 | -0.2± 2.9 | -0.2± 1.6 | -0.7± 10.6 | 1.0± 9.1 | 0.0± 12.4 | -0.2± 2.7 | -0.1± 1.2 |
| Normal BMI (≤ 24.9 kg/m2) | -2.7± 6.9** | 0.8± 7.9 | 1.7± 10.9 | 0.2± 3.3 | 0.0± 2.7 | -1.0± 7.6 | 2.7± 8.4* | -1.5± 9.9 | 0.1± 3.0 | -0.3± 1.4 |
| Overweight (25- 29.9 kg/m2) | 0.1± 8.5 | -1.1± 9.0 | 1.1± 11.1 | -0.1± 3.4 | 0.1± 2.0 | 1.8± 9.4 | 0.3± 8.8 | -1.5± 14.2 | -0.4± 2.7 | -0.2± 1.3 |
| Obese (> 29.9 kg/m2) | 1.1± 10.4 | -0.8± 7.8 | 0.1± 12.7 | -0.4± 2.3 | 0.0± 1.0 | -0.9± 12.1 | 0.8± 7.6 | 0.4± 11.5 | -0.3± 2.0 | 0.1± 1.0 |

Wilcoxon Signed Ranks Test * p <0.05, **p <0.01, MD- mean difference, SD- standard deviation. Glucose metrics presents, % time above the range in level 1 (TAR-LI)> 13.9 mmol/L and level 2 (TAR-L2), 10.1 – 13.9 mmol/L; % time in range (TIR), 3.9–10 mmol/L; % time bellow the range in level 1 (TBR-L1), 3.0 -3.8 mmol/L, and level 2 (TBR-L2) < 3.0 mmol/L.
